# Pediatric Patients Treated for Leukemia Back to School: A Mixed-Method Analysis of Narratives about Daily Life and Illness Experience

**DOI:** 10.3390/bs10070107

**Published:** 2020-07-01

**Authors:** Marta Tremolada, Livia Taverna, Sabrina Bonichini, Marta Pillon, Alessandra Biffi, Maria Caterina Putti

**Affiliations:** 1 Department of Developmental and Social Psychology, University of Padua, Via Venezia, 8 35131-Padova, Italy; s.bonichini@unipd.it; 2 Pediatric Hematology, Oncology and Stem Cell Transplant Center, Department of Woman’s and Child’s Health, University of Padua, 35128 Padua, Italy; marta.pillon@unipd.it (M.P.); alessandra.biffi@unipd.it (A.B.); mariacaterina.putti@unipd.it (M.C.P.); 3 Faculty of Education, Free University of Bozen-Bolzano, Brixen-Bressanone, Viale Ratisbona, 16 39042-Bressanone, Italy; livia.taverna@unibz.it

**Keywords:** pediatric leukemia, narratives, peer relationships, school re-entry, follow-up perceptions

## Abstract

In the last few years, more children and adolescents healed from leukemia go back to their daily life, even if they can show some psycho-social difficulties. The study adopted semi-structured interviews and a mixed-method approach to examine the narratives of 75 children and adolescents about their return to school post 2-years treatment for leukemia. The aims are to collect their illness experiences, to understand how they feel about school and daily routines and to identify the best socio-demographic and illness predictors of a good re-adaptation to school and daily life. The results show that by increasing age and when the pediatric patient have received a hematopoietic stem cell transplantation, at the stop-therapy time, her/his perception about relationships at school and academic performance decrease, especially if his/her feelings about the disease and follow-up visits are negative.

## 1. Introduction

The path that the pediatric patient with leukemia faces is temporally marked by different phases, which correspond to different stages of the disease and which can end with the remission phase. In this phase, the anxiety related to the disease is reduced, until the interruption of treatments (stop therapy), which represents a moment of positive, but very delicate transition. For leukemia, stop therapy occurs two years from the time of diagnosis, and means that the highest risk period for a relapse has been passed. The treatment protocol and the intake of cancer drugs are interrupted, and from this moment, periodic checks to monitor health will begin, lasting for the next five years. This is a period of surveillance, during which the child can return to normal social life.

In the literature, there is a distinction between near-term survivors and long-term survivors, to identify the two moments described above. Cantrell and Conte [1] define the former as having reached the end time of therapy following the complete remission of the disease, but that still not having accomplished the five-year reference period in order to be considered cured, and being included in the category of long-term survivors. In general, the term “survivor” refers to those patients who have been diagnosed with leukemia, but who have currently finished all medical treatments for at least six months [2].

Despite the large number of children who manage to recover from leukemia, an ever increasing quantity worldwide [3], little research has been conducted on the psychological and social aspects of children or young people who have recently completed the treatments. In fact, the literature has investigated more the effects of long-term survivors, neglecting the moment of transition from stop therapy to off therapy, and even examining only adult patients [1].

Pre-adolescence and adolescence are delicate periods of transition and change, in which the individual experiences a growing sense of independence from the family. In hospitalized children, this normal evolution seems to stop, and instead there is a strong dependence on the adult world, on parents and doctors. The fact of having a tumor, with its treatment and hospitalization, means that these children are deprived of the opportunity to plan their future and to continue their normal emotional development towards adulthood. Younger patients (aged 8–13 years) have been reported to be more at risk than older ones in their problems’ intensity and psychological symptoms during the first 6 months of treatments, with females and Acute Myeloid Leukaemia patients showing lower current life satisfaction perceptions [4]. However, this experience can also allow them to acquire earlier a high level of maturity: adolescents with a tumor must make decisions and take on responsibilities that force their developmental trajectory towards an untimely adult maturity, which is generally achieved gradually, thanks to the normal daily experiences in adolescents [5].

With the completion of the therapies, these children find themselves in a new world, for which they have not been prepared, and must try to find their place. As Cantrell and Conte [1] point out, this paradox is also strengthened by the fact that they can physically appear healthy, without identifiable treatment signs. In reality, the lack of side effects or particular physical signs does not necessarily correspond to emotional well-being. One of the main tasks that the adolescent must face is to know and accept their new identity, with the awareness that it will no longer be as before. The adolescent’s goal is no longer to face the disease, but to know the new self and look beyond. Some teenagers express the need to redefine the concept of “normal life” and the need for support during this period, showing difficulties in reintegrating into school life and in making new friends. Others, however, describe feelings of guilt for being healed, towards those who have entered the terminal phase and for involving family and friends in their experience [6]. There is often an ambivalence in their feelings: the completion of treatment represents the end of a long and tortuous experience, but is also a moment of crisis that can lead to the development of anxiety and stress [1].

In research conducted by Von Essen et al. [7] on Swedish adolescents within five years of stopping therapy, it emerged that the first period following the end of the treatments is characterized by an increased risk of reduced psychosocial functions, compared to the treatment period. The fact that patients during treatment are supported by doctors, nurses and parents partly explains the lower stress levels, compared to the following period. In fact, when they no longer receive regular and daily support, some difficulties may begin to be evident. This study shows that patients whose diagnosis was made in late childhood or adolescence are more at risk of developing problems than those who experienced the disease in the early years of life. Different results emerged in a research conducted by Maggiolini et al. [8], in which a group of pediatric cancer patients aged between 12 and 20 years, off therapy for at least two years, was compared with a group of healthy peers, to investigate the concept of self in relation with psychosocial, sexual, family and coping aspects. It emerged that the former had greater confidence in dealing with adolescent problems, greater emotional stability and a positive image of themselves and their body, a good relationship with parents and a greater appreciation of the support received from them. The authors motivate these results by referring to the concept of “personal growth”, which helped them to see life in a positive way after such a difficult experience, as other studies pointed out [9].

Although it is actually possible to continue school activities in the hospital, re-adaptation to the school environment is particularly difficult for some patients, both in the learning and relational areas, declaring low social support than healthy peers [9]. The ex-patients show a tendency to isolation, a higher percentage of failures and a greater chance of being bullied [10]. Even though more than half of the subjects describe school as a positive experience [11], many children show nervousness, due to lack of trust [12], concern and a sense of diversity compared to peers [6]. In a cross-sectional study, which used both a qualitative and a quantitative approach, on 137 children and adolescents aged between 9 and 16 years, Li et al. [2] identified, with regard to physical symptoms, some recurring themes, such as loss of memory, poor concentration and an increase in fatigue, the inability to participate in the same recreational activities as before, due to low physical strength, with consequences in terms of social relations. Another emerging aspect is the school performance: the patients reported that they had to make greater efforts to obtain previous results, and to recover what they have lost during hospitalization. The healed patients stressed that the greatest psychological support was that from the parents and siblings, who made them feel less alone in this experience. In an attempt to rebuild a social life, children tend to present themselves in a more positive perspective and show less antisocial behavior. Despite this, they reported being unsatisfied with their friendships and having some difficulties interacting with others, unlike peers without any past illness [13]. This view was also confirmed by the reports of parents and teachers: the former reported that their children had no close friends to confide in; the latter argued that these students were less popular than the others within the classroom context, and that they were rarely chosen as the first option for play and group activities by their classmates. The study by Foster et al. [14] examined the sense of personal efficacy of 56 ex-patients aged between 11 and 20 years, an aspect rarely taken into consideration in the literature. Self-efficacy is a complex construct that influences an individual’s commitment to achieving their goals, as well as motivation and resilience. It also refers to the everyone’ beliefs about their ability to succeed in school. In adolescents, school self-efficacy is predictive of academic achievement, just as feeling competent in relationships with others is related to good adaptation [15]. Since treatments for leukemia involve some changes in social relationships, it implies the interruption of the normal school path and an increase in vulnerability, it can be supposed that adolescents who finish their therapies may find it difficult to develop or maintain a positive perception of their abilities and their future. The age at the time of diagnosis and the time passed since the interruption of the therapies were not associated in this study with the perceived vulnerability and self-efficacy. The teenagers in this sample showed a lower self-efficacy in the social area than healthy peers, in accordance with other studies that reported difficulties in developing and maintaining social relationships and with a partner [16].

This study is part of a larger research project entitled “Predictive family factors of adaptation and short and long-term quality of life in children with leukemia and their parents. A longitudinal study”, carried out at the Pediatric Onco-Hematology department of the hospital of Padua, subject to authorization by the Ethics Committee for the experimentation of the Padua Hospital. The research is divided into several sub-projects, the general purpose of which is to evaluate the child’s adaptation to leukemia, the short and long-term implications of the disease, which areas of development can be more limited and which factors may support better adaptation. It is a longitudinal and multi-method analysis that examines families from the moment of diagnosis to stop therapy and in some intermediate stages of the treatment period. This research report refers to in-depth interviews with children and adolescents at the time of stopping therapy.

Based on what has been reported by the examination of the literature compared to the time of the stop therapy, it is possible to formulate some research questions and hypotheses:

1. How do ex-pediatric patients perceive the experience of illness and their daily life, especially with regard to the relationship with the school and the relationships with classmates and teachers? (research question 1)

**Hypothesis** **1a.** *Although we expect the ex-patients not falling into real psychopathological frameworks [8], we believe that they may declare difficulties related to the specific aspect of social reintegration [1,14].*

**Hypothesis** **1b.** *Although ex-patients may show a good perception of the relationship with the school [11], we expect that they could highlight relational problems with classmates and teachers related to their performance [10,16].*

2. What are the socio-demographic factors, related to the disease and the experiences of stop therapy, that may influence the perceptions of ex-patients on the relationship with the school and on adapting to daily life? (research question 2)

**Hypothesis** **2a.** *We hypothesize that the higher age of the ex-patients corresponds to worse scores in their perception of life and in relationships with the school [17], with the females reporting greater difficulties and with the high social class that positively influences adaptation [18].*

**Hypothesis** **2b.** *Ex-patients who have been treated for Acute Myeloid Leukemia (AML) and have undergone hematopoietic stem cell transplantation experience greater psychological suffering and a lower level of school adaptation.*

**Hypothesis** **2c.** *Conflicting data concern the influence of the “age at diagnosis” factor on school and social re-adaptation, ranging from no impact [14], to a greater risk for those who fall ill in adolescence [7]. It is therefore a matter of assessing whether there is influence of this factor and in which direction.*

**Hypothesis** **2d.** *How ex-patients experience the moment of stop therapy in which they no longer receive regular and daily support from health professionals could influence their social and educational well-being [7].*

## 2. Materials and Methods

The participants in this study are 75 children and adolescents (38 males and 37 females), with an average age at diagnosis of 11.10 years (SD = 4.06; range: 5.5–19.33) and at the assessment of 13.10 years (DS = 4.06; range: 7.5–21.33), under treatment at the Pediatric Oncohematology Clinic of the Padua Hospital. Of these, 65 were treated for acute lymphatic leukemia and 10 for acute myeloid leukemia, with more intense therapy and a worse prognosis. All patients of school age, Italian native speakers and already participating in the previous evaluation phases of the entire research project, were contacted personally or by telephone. The majority of patients (74.66%) joined the study, except 6 who relapsed, 2 who died, 4 who are checked in another treatment center and 7 who, despite having agreed to participate in the research, were not interviewed because of the overlap with other visits.

Table 1 and Table 2 show the socio-demographic and medical information of the young patients and their parents respectively, obtained through a questionnaire completed by the parents at the stop therapy and the medical records.

### 2.1. Ecocultural Family Interview-Cancer Young Patient Version

The Ecocultural Family Interview (EFI)-Cancer (EFI-C) is a parent interview, which explores the daily routines of family life and the salient concerns regarding how that routine is organized. The EFI is not a question-answer formal interview, but it assumes more a sociolinguistic form of an everyday conversation about daily life. The interview is a mix of conversation, probing questions by the interviewer and preplanned questions. When patients or their families talk about their everyday life and routines, they spontaneously talk about all their adaptation tasks, script or set of actions, beliefs and motivations stored in their memory. In each version of this tool there are: dimensions that investigate the daily life of the family (the prevalent way in which family members organize their daily activities and activities, based on their resources), the way in which the person interviewed relates to the entourage that surrounds him (for example extended family, friends, neighbors, medical personnel) and the overall meaning that it is given to one’s daily actions. There are also specific dimensions for the context of the population studied. The EFI-Cancer (EFI-C), developed by Tremolada, Bonichini, Weisner, Basso and Pillon [19] in collaboration with the Department of Psychiatry, UCLA, USA, makes it possible to study the family context and the adaptation of affected children from tumor pathology, according to the narratives carried out by the parents.

In the present study, a new version of the Ecocultural Family Interview-Cancer (EFI-C) was used, adapted, in agreement with the creator of the tool Thomas Weisner, to be applied to young patients at the stop therapy time. The psychologist principal investigator started the interview by asking the patients to tell about their life, focusing on the following aspects: daily activities, the relationship with classmates and teachers (period of reintegration, performance, difficulty), friendships, plans for the future, the relationship with their own body, with family members and hospital experience. Through the patient story, the interviewer becomes aware of qualitative data on adaptation and psychological well-being at this delicate phase.

The interviews lasted a little less than about one hour, were transcribed verbatim and then used to develop items which represented meaningful aspects of these patients’ lives. After reaching overall agreement on these topics and themes, we developed specific items, using examples drawn directly from the interviews for each of the items, capturing the variability in patients’ views across the sample; these items were then coded. The variability in each EFI item is expressed by a range from 0 to 8, so we found at least one example for each score level of each item: Low (0,1,2); Medium (3,4,5); High (6,7,8). Two independent judges then scored the interviews using the codebook. During this process, the disagreements between the judges were resolved through team discussions. The items have been organized in such a way that a higher score also indicates a better and more positive perception by the ex-patient of the aspect considered, while a low level indicates important difficulties in this area. The researchers’ work involves carrying out thematic analyses and creating quantitative scores through the construction of a coding system (codebook), and has made it possible to establish the young patient version, consisting of 53 items that identify the dimensions treated in the interviews and more studied in the literature. These specific items were extracted from the patients’ narratives by the entire research team in group discussions. The single items were grouped into big psychometric valid and coherent dimensions which were tested for coherence and reliability. Above each EFI-C theme, we reported its internal coherence and the number of items of which it was composed. Table 3 presents the items to code the interviews of children and young people with leukemia at stop therapy, grouped by size: a first group of codes refers precisely to the time of stop therapy and investigates, for example, current well-being and fear of checks; the second dimension concerns the school environment; other items explore patients’ daily activities and their friendships; still others are specific to the experience lived in the hospital and concern the relationship with the medical staff, the adaptation to the procedures, the friendships established with other patients; another dimension focuses on perceived physical well-being, on acceptance of one’s own body; finally, the last items investigate aspects related to the family, cohesion and perceived autonomy. In the codebook there are exemplary interview excerpts of each level for each item that will guide the researcher in the work of coding the interviews.

In this study, the dimensions and the respective items derived from the narrative interviews relating to their relationship with the school and lives in the moment of interruption of the therapies and towards the disease will be illustrated. The number of questions was variable, the technique used a more thematic approach to apply the questions, and the frequency of questions depend on subjective characteristics, behaving such as a conversation, rather than a formal question–response interview.

Table 3 shows the 6 dimensions in which the items have been grouped: current experience on stop therapy and illness (5 items), relationship with school (7 items), daily life and social relationships (7 items), relationships with the clinic during illness (6 items), relationship with body and central venous catheter (5 items), relationships with family (6 items). Cronbach’s alpha for each dimension ranges from a minimum value of 0.64 (relationship with body and the Central Venous Catheter (CVC)) to a maximum value of 0.84 (relationship with school), values considered discrete and good. For data encoding throughout the codebook, the degree of agreement between two independent judges was assessed by means of the Cohen kappa coefficient, from which a value equal to 0.74 emerged and through the Spearman correlation equal to 0.96, both indices of good agreement of the encodings. All cases in which the two judges had not reached an agreement were reviewed, leading to a unanimous coding used for data analysis.

### 2.2. Socio-demographic Questionnaire

The socio-demographic information on the family was obtained from the questionnaire completed by parents at the stop therapy: data relating to age, parents’ schooling, working, economic and housing conditions, the composition of the family were used.

### 2.3. Medical Records

Strictly medical information, such as the type of leukemia, the number of days of hospitalization, the hematopoietic stem cell transplantation in the treatment protocol, were taken from the patient’s medical records, subject to authorization by the families of the patients involved.

### 2.4. Procedure

The EFI-C in-depth interviews of the young patient version, lasting up to one hour, were audio-recorded, subject to authorization by signing the informed consent from the parents, on the day of stop therapy or, when not possible, in a next moment during the patient check-ups, always within a month from the end of the treatments. Pediatric patients had already resumed school activities, although not yet with full attendance. These interviews were conducted without the presence of the parent in a quiet room inside the Day Hospital of the Pediatric Oncohematology Clinic of the Padua Hospital. The clinical psychologist and researcher who conducted the interview already knew both the pediatric patient and the family members. In some cases, a co-host was present who silently observed the interview and asked any questions for clarification at the end. The first question asked to patients was to describe how a typical day unfolds, from morning to evening, starting from a broad topic, and then reaching some predefined themes: the relationship with the school, with the family, with the hospital, perceived well-being, expectations for the future. A fundamental prerequisite for this conversation is empathic listening, which allows the interlocutors to be free to tell each other, to express their feelings, with the fears and hopes that accompany them [20]. This approach is fundamental for having information valid and reliable from the direct voices of the children and adolescents healed from leukemia. In-depth narratives also belong to a positive approach that identifies story-telling as the more trustable methodology to obtain free and naturalistic information on children and adolescent experiences, in order to identify their needs and take care of them.

### 2.5. Statistical Analysis Plan

Preliminary qualitative and next descriptive analyzes on the contents of the interviews were carried out first, to answer to the first research area regarding how ex-pediatric patients perceive the experience of illness and their daily life, with a particular attention to the relationship with the school and the relationships with classmates and teachers.

Pearson’s correlations between the continuous independent variables (age at diagnosis, age at evaluation, years of schooling of parents, average working hours of parents, lived on illness and stop therapy), or categorical at two levels (e.g. gender, type of leukemia) and the dimensions obtained from the EFI-C version of young patients (relationship with school, daily and social life). ANOVAs were also conducted on the dimensions of the EFI-C, with independent variables the socio-economic status and the number of siblings. Based on the results of the correlational analyzes, hierarchical regression models are proposed, to identify the socio-demographic and medical predictors of a good adaptation to school, and to the daily life of young patients to answer the second research question, with associated hypotheses about which socio-demographic, disease and the experiences of stop therapy factors may influence the perceptions of ex-patients on the relationship with the school and on adapting to daily life.

## 3. Results

### 3.1. How Do Ex-pediatric Patients Perceive the Experience of Illness and Their Daily Life, Especially with regard to the Relationship with the School and the Relationships with Classmates and Teachers?

To answer research question 1, on how children and young ex-cancer patients perceive the experience of disease and their daily life at stop therapy, descriptive analyses of the dimensions reported in the codebook and presented below were carried out, calculating the means and standard deviations (Table 4). As the score increases, the satisfaction of the child or adolescent on that particular aspect is higher, but, since they are not standardized on other clinical or healthy populations, these scores cannot give indications on psychopathological aspects.

With respect to the first hypothesis on adaptation (1a), results show that only a minimum percentage of the participants (13.2%) is placed in the worse level (below the score of 3) of perceived satisfaction with “relationship with the school ”, while the majority (46.66%) are in the moderate score between 3 and 5. Seven children (9.33%) show difficulties in their “experiences related to disease and stop therapy”. As for the aspect of social reintegration and readjustment to daily life (“daily life and social relationships”), 14.76% declare important difficulties and 74.66% report slight difficulties. Hypothesis 1a is therefore confirmed. These children and young people can feel isolated and “different” from other peers, especially in pre-adolescent age, a delicate aspect very well expressed by the words of an 11-year-old girl “I would like to be just a little like the others ... normal (...) I feel normal, it’s not that I feel different, but I want to be like the others (...) because I feel back in school ... it’s hard to make friends ... ”.

By examining, in detail, the specific items about the school to test Hypothesis 1b, 11.2% report low scores on school approval, while 29.1% place at moderate scores and most declare a good rating (40.3%). Children report lower scores (15.1%), or moderate (28.3%), on their academic performance and physical adaptation to school (26.92% low and 36.53% moderate respectively). Instead, they declare good relations with their classmates (47.54%) and regular attendance at school regularly without too many absences (60.31%) in most cases. Hypothesis 1b is only partially confirmed. For example, on physical adaptation to school, a 17-year-old boy tells us about the beginning of school: “I remember that at the beginning, it was also stress then oh well at the beginning of chemotherapy so the body was not accustomed to nothing, I also had memory lapses, because I didn’t, I did something and maybe I didn’t remember it afterwards”. The perceptions of children and young people related to their body are on the same line, as can be seen from the words used by this 17-year-old girl: “during the therapies I didn’t pay attention to it, however now .. that is, like stretch marks that I have one tide and already having stretch marks makes me nervous, ... sometimes I say: I want to be thinner (...) so it is not that I mean now diet I don’t eat now, no, I continue to eat (...) when I’m nervous then I eat (...) er but yes I have these moments that I say “I want to be thinner” and I suck ... ”

Regarding the dimension of “relationships with the family”, the ex-patients above all underline positive aspects. For example, a 19-year-old girl tells her experience with her mother like this: “she (mum) was in symbiosis with me. She experienced joys and sorrows with me ... as if she had been the sick. Here ... it’s something that has helped me, it still gives me security, seeing a shoulder alongside, strong ... ”.

### 3.2. What Are the Socio-demographic Factors, Related to the Disease and the Experiences of Stop Therapy, That May Influence the Perceptions of Ex-Patients on the Relationship with the School and on Adapting to Daily Life

The analyses of variance that were conducted to evaluate whether there were differences in the adaptation capacities of the ex-patients with respect to the socio-economic condition (partly Hypothesis 2a) did not reach any significance.

To assess the possible hypotheses of this research question (hypotheses from 2a to 2d), Pearson’s correlations were carried out to evaluate the possible associations between the socio-demographic characteristics, the disease of the participants and the dimensions investigated (relationship with the school, current life at stop-therapy, daily life and social links) (Table 5).

From these analyses, it emerges that the medical variables “type of leukemia” and “transplant” in the treatment are significantly correlated to the dimensions of the relationship with the school and the relationships with the care staff during the illness. As far as socio-demographic variables are concerned, age is correlated negatively with the “relationship with school” dimension and positively with “relations with the clinic during illness” one. Finally, the parent’s average working hours are negatively associated with the “family relationships” dimension.

Based on the results of the correlations, hierarchical regression models are run, starting from the associated factors that have obtained a significant value. This analysis allowed to understand which factors influenced more our dependent variables, relationship with school and physical adaptation to school. In the first model, which has as its dependent variable the “relationship with the school”, the following variables have been included in the first block: age of the child, gender, diagnosis of the child/adolescent (acute lymphloblastic leukemia vs. acute myeloid leukemia) and transplant of hematopoietic stem cells (no/yes), while the variable current life of the child on stop therapy and on disease has been inserted in the second block. The second model explains most of the variance (R^2^ = 0.52; F = 16.98; *p* = 0.0001), where the minor age (β = −0.35, *p* = 0.0001), the not undergoing hematopoietic stem cell transplantation (HSCT) (β = -0.28, *p* = 0.02), and the presence of positive experiences towards disease and stop therapy (β = 0.46, *p* = 0.0001), positively influence the relationship with the school.

**Figure 1:**
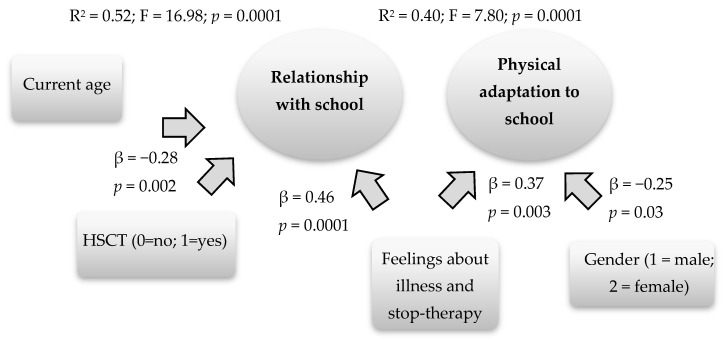
Predictive factors of the relationship with the school and of the physical adaptation to the school.

As many models were created with the same independent variables and with dependent variables the respective seven items of the global dimension “relationship with the school”. The individual items are predicted by the same variables of the global dimension, except for the item related to physical adaptation to school, which is also predicted by gender, with the females adapting more (Figure 1).

## 4. Discussion

This study focused on the data that emerged from the interviews with young patients at the time of the stop therapy, which occurs two years after the onset for leukemia. Often, research in this field has dealt with patients still in therapy or those out of therapy, the survivors, without however distinguishing them based on the time elapsed since the time of the stop therapy [3], considering all the different diagnosis of cancer in children and through self-questionnaires and proxy-reports.

This study used the in-depth interview technique, which allows us to have more information on the possible needs and adaptation to the daily life of ex-patients treated for leukemia, compared to the administration of the questionnaires. The emerging literature generally focused on the qualitative approach to illness narratives adopting narrative inquiring, which is the study experience conceived as a story, but above all it is a specific way of thinking experience [21] (p. 477). Another adopted methodology with childhood leukemia survivors can be the grounded theory, a method that allow researchers to reconstruct the “theory” that the participants have developed with respect to a certain phenomenon/process [22]. This methodology considered data analysis as a process that does not depend exclusively on data characteristics (i.e. narratives), but also on research questions and, consequently, on the selected research method. Then, there is also the narrative dialogic analysis adopted with palliative cancer patients [23], where the coding strategy revolves around reading and re-reading each single interview from the viewpoint of the person him or herself, and to provide insight into how people confer meaning to their experiences.

In this study, we used another approach that derived from the ecocultural theory. The Ecocultural Family Interview is based on the theory that by using the patients’ own categories and stories, with the themes and topics embedded in them, the researcher gets closer to the parents’ points of view and experiences [19]. This mixed-method approach adopting in depth interviewing applies a positivist point of view considering not relevant the pathological aspects/sequelae of leukemia, but principally the meaning they could give to their illness experience. This is identified the best strategy for that project, giving voices of the direct experiences of children and adolescents. Their opinion is crucial to understanding their needs, and to ameliorate the intervention given by health professionals. Through this mixed qualitative–quantitative analysis, it is possible to deepen the implications at a psycho-social and scholastic level at the time of the end of the therapies.

Responding to the first area of investigation about how ex-pediatric patients perceive the experience of illness and their daily lives, we examined each interviews’ dimensions’ scores. Firstly, analyzing the scores obtained about their relationship with the school and the relationships with classmates and teachers from the interviews, it emerges that the school represents an important and fundamental link with the “normal” world, even after the therapies are finished. As shown by the studies in the literature, they report satisfaction with relations with the school, this is a positive result, even if they underline the difficulties in the aspect of social reintegration both in the school context and in daily life. There is a sort of ambivalence in their feelings as reported in precedent studies, probably due to the subjective experience of each patient regarding the experience of returning to schooling [6]. In this regard, it would probably be necessary that health professionals and hospital teachers communicate more efficaciously with the original school system, in order to empower communication during the therapies and to set up specific and ad-hoc re-entry programs.

Then, regarding the current perceived family support and relationships, we found that ex-patients give good assessments of their family relationships, from which they feel very supported, as confirmed by other studies [7,8]. Additionally, the satisfaction of the relationship with one’s own body and the management of the central venous catheter that the children had to keep until the stop of the therapies is also low or moderate, as pointed out in other studies [16]. It could be important to give support to children and adolescents in their body perceptions, in order to increase their acceptance of possible temporary or definitive body changes.

To answer the second main research area regarding which socio-demographic and illness factors could impact the perceptions of ex-patients on the relationship with the school and on adapting to daily life, we first examined gender. From the point of view of physical adaptation to school (concentration, headache, tiredness), the difficulties are more reported in boys than in girls [2]. Male patients are probably less interested in investing their time in school, preferring other activities such as sport/music/being with friends and, therefore, they could show greater intolerance than girls, as can be seen from the studies on healthy population [9].

Secondly, we also took into consideration current age, and the results showed that by patient’s increasing age, his/her relationship with school perception and academic performance deteriorate at the stop therapy time. Child and teenagers face numerous challenges during their growth. The transition from primary school to first grade secondary school, for example, can affect academic performance, due to the increased workload required and the level of inclusion, as well as the relationship with classmates.

Thirdly, also the disease/treatment factors could impact on their school re-adaptation, with our results showing that children that have undergone hematopoietic stem cell transplantation (HSCT) are identified as being more at risk of a problematic school re-integration. Children and young people with high-risk myeloid or lymphatic leukemia who have undergone transplant treatment spend a large part of the period of the disease in hospital, even in the isolation area, and have been subjected to more intense therapies. All these aspects could provoke a low school attendance and the possibility of having greater attention and concentration difficulties due to therapies, with the possibility of the onset of ADHD [24].

In addition, another possible factor that could impact on their re-adaptation to school is how they perceived the stop-therapy phase. If young patients experience it more serenely, they will also have less psycho-physical problems in their adaptation to school. The experiences and possible fears of the disease returning (with states of excessive emotional activation) could negatively influence their ability to concentrate, increasing fatigue. Furthermore, if the young patient has recurrent traumatic thoughts about the illness experience and how the disease has affected his/her life in a negative way, the perception of school and the relationship with teachers and classmates will not be positive. It is important that they are able to talk with health professionals about their feelings, doubts, worries or other questions about this delicate phase of therapy interruption.

As can be seen from these empirical results, the patients report a positive point of view in all the factors considered, especially concerning the level of school satisfaction and school placement, while the aspects with the lowest scores are related with physical adaptation to school rhythms. The wide individual variability, evidenced by the high standard deviations, also affects these data.

## 5. Conclusions

The use of a semi-structured interview, such as the Ecocultural Family Interview-Cancer (EFI-C) young patients version, is very important in the context of pediatric psycho-oncology, as it allows the psychologist to establish a relationship based on trust, so that the child/boy feels free to express himself, and to express any doubts or concerns. It makes it possible to obtain a wealth of qualitative data, and, at the same time, to be able to transform them into quantitative data. Furthermore, it seems to give a more detailed and valid picture of the post-illness situation, given that the aspect of social desirability is lacking, which can condition the responses of the children in the self-report questionnaires.

However, this type of search tool has some limitations, such as the risk of inducing certain answers through the questions asked, or the influence that the interviewer may have on emotions and, consequently, on answers. It should also be considered that, by carrying out the transcription work of the interview, however scrupulous and detailed it is, there is the possibility that some information may be lost, especially of a non-verbal type.

The research could continue by completing the collection of data through teachers, with information relating to the behavior of ex-patients at school and the relationship with classmates, to investigate the aspect of social reintegration. Based on the results obtained and the literature on the delicate moment of the return of children and young people to school at the end of the leukemia therapies, it is possible to discuss useful applicative ideas for teachers who welcome their pupil back into the classroom.

We have already highlighted the problem of the scarcity of studies concerning the end of therapies, despite the evidence of a crucial moment for the young ex-patient. At this phase, he/she has a role to resume, at least in part, the life abandoned at the time of diagnosis, but in a psychophysical condition different to that in which he was before the disease. The period of hospitalization causes an interruption or weakening of social relationships, especially with peers; the therapies bring bodily changes that often do not make it possible to resume the same activities that took place previously. The return to school must be addressed by taking into account the parents’ apprehension about health, and often also their fears about it: leaving the clinic, fears arise due to relapses or other problems. It is therefore important to encourage research in this area, to plan projects to improve this delicate transition, from the end of treatment, to the resumption of all activities of daily life, where school is one of the most significant.

The transition from the end of treatment to the return to daily life can be investigated through quantitative and qualitative research, which allows us to grasp different aspects and, if well integrated, may offer an exhaustive picture of the psychosocial consequences of the experience of childhood cancer. Quantitative research and standardized tools allow the examination of cause-effect relationships, but leave little space for understanding the experiences, an aspect that is mostly captured by qualitative methods. The latter consider the point of view of the young ex-patients through their direct story about thoughts, emotions and experiences. If the presence of a problematic adaptation can be detected from the quantitative approach, it is thanks to the qualitative one that the individual aspects can be analyzed and understood in greater detail.

## Figures and Tables

**Table 1:** Patients’ socio-demographic characteristics.

		Mean	SD
Age at diagnosis		11.10	4.07
Age at assessment		13.10	4.07
		Frequency	%
Gender	Male	38	50.7
	Female	37	49.3
	Total	75	100
Age bands at diagnosis	5–7 years old	10	13.3
	8–11 years old	36	48.0
	>11 years old	29	38.7
	Total	75	100
Age bands at assessment	7.5–11 years old	24	32
	11.01–14 years old	24	32
	14.01–21.33 years old	27	36
	Total	75	100
Type of leukemia	ALL	65	86.7
	AML	10	13.3
	Total	75	100
HSCT	No	64	85.3
	Yes	11	14.7
	Total	75	100
Number of siblings	None	18	24
	1 sibling	43	57.3
	2–3 siblings	12	16
	Total	73	97.3
	Missing	2	2.7
	Total	75	100

**Table 2:** Participants’ education and their family characteristics.

		Frequency	%
Educational level	5 years of schooling	1	1.3
	8 year of schooling	25	33.3
	13 years of schooling	37	49.3
	First degree	11	14.6
	Missing	1	1.3
	Total	75	100
Mean job hours/weekly	0	6	8
	5	7	9.3
	15	4	5.3
	25	15	20
	35	23	30.7
	45	12	16
	50	4	5.3
	Missing	4	5.3
	Total	75	100
Perceived economic condition	Low	16	21.3
	Medium	43	57.3
	High	14	18.7
	Missing	2	2.7
	Total	75	100
Home situation	Rent	13	17.3
	Home ownership with mortgage	21	28
	Home ownership without mortgage	35	46.7
	Other	3	4
	Missing	3	4
	Total	75	100

**Table 3:** Cronbach explanation, item grouping and Alpha values for Ecocultural Family Interview **(EFI)**-Cancer (EFI-C) dimensions.

Dimension	Item	N Item	Alpha
Current life of the child on stop therapy and on disease	- level of perception of the general well-being / serenity of the child at the time of the stop therapy - level of concern related to controls - level of enthusiasm for life - level of perception of normality of one’s life ability to remember positive events related to the experience of illness - level of influence of the disease in the perception of one’s life	7	0.72
Relationship with school	- level of appreciation of the school - general level of school placement - welcome level of teachers and classmates at the time of school reintegration - relationship with teachers - relationship with companions - level of academic performance - physical adaptation to school	5	0.84
Daily life and social relationships	- level of physical activity carried out by the child - level of recreational activities carried out by the child - level of planning of future activities - quantity or quality of friendships declared by the child - satisfaction level of relationships with friends - preference level of a friend in particular - pre-illness social network maintenance	7	0.76
Relations with the clinic during the illness	- relationship with doctors - child’s preference level for some doctors/nurses - relationship with nurses - report with the teachers of the department - level of socialization in the hospital - level of attachment to the hospital environment	6	0.72
Relationship with body and CVC	- level of acceptance of medical procedures and drugs and their possible side effects - level of perception of negative events during the experience of illness - level of desire to remove the CVC - ability to remember the moment when they removed the CVC - level of perception of normality following the extraction of the CVC	5	0.64
Relations with family	- level of agreement between brothers - level of support perceived by the family - level of support perceived by the mother - level of support perceived by the father - level of agreement with the mother - level of agreement with the father	6	0.83
		36	

**Table 4:** Descriptive statistics of the dimensions of codebook referred to the child at stop therapy.

Dimensions and items	Mean	SD
Current feeling of the child on stop therapy and on disease	4.81	1.25
Level of perception of the general well-being / serenity of the child at the time of the stop therapy	5.86	2.03
Level of control related concern (turned over)	4.64	2.82
Level of enthusiasm for life	5.40	1.88
Level of perception of normal life	5.05	2.09
Ability to remember positive events related to disease experience	3.64	2.10
Level of influence of the disease in the perception of one’s life	3.74	2.26
Relationship with the school	5.11	1.78
Level of appreciation of the school	5.37	2.18
General level of school placement	5.35	2.46
Degree of reception of teachers and classmates at the time of school reintegration	4.47	2.21
Relationship with teachers	4.55	1.73
Relationship with comrades	5.26	2.18
Level of academic performance	5.30	1.96
Physical adaptation to school	4.32	2.22
Daily life and social relationships	4.5	1.36
Relations with clinical staff during the illness	4.44	1.83
Relationship with the body and CVC	4.19	1.71
Relations with the family	6.27	1.47

**Table 5:** Correlation matrix between the codebook size and patient’s socio-demographic variables.

		Age of Child in Months	Child’s Gender	Child’s Diagnosis	HSCT (No/Yes)	Parental School Years	Mean Hours/Weekly
Current life of the child on stop therapy and on disease	Pearsons’ correlation *p* N	−0.16 0.18 75	−0.15 0.21 75	−0.01 0.91 75	−0.19 0.11 75	0.02 0.84 74	−0.13 0.26 71
Relationship with school	Pearsons’ correlation *p* N	−0.49 ** 0.001 75	−0.17 0.14 75	−0.30 ** 0.01 75	−0.45 ** 0.001 75	0.13 0.27 74	0.20 0.10 72
Daily life and social relationship	Pearsons’ correlation *p* N	0.001 0.99 75	0.05 0.66 75	0.09 0.43 75	−0.04 0.72 75	−0.20 0.08 74	0.04 0.73 71
Relations with Clinical staff during illness	Pearsons’ correlation *p* N	0.57 ** 0.001 73	0.18 0.12 73	0.30 * 0.01 73	0.30 ** 0.01 73	−0.14 0.23 72	0.02 0.87 69
Relations with body and CVC	Pearsons’ correlation *p* N	0.23 0.53 73	0.18 0.13 73	0.20 0.10 73	0.18 0.12 73	0.12 0.32 72	−0.13 0.30 69
Relations with family	Pearsons’ correlation *p* N	−0.14 0.23 72	−0.13 0.28 72	0.02 0.85 72	0.11 0.33 72	−0.07 0.55 71	−0.36 ** 0.001 68

* *p* < 0.05; ** *p* < 0.01.

## References

[1] Cantrell: M.A., Conte T.M. (2009). Between Being Cured and Being Healed: The Paradox of Childhood Cancer Survivorship. Qual. Health Res..

[2] Li H.C.W., Lopez V., Joyce Chung O.K., Ho K.Y., Chiu S.Y. (2013). The Impact of Cancer on the Physical, Psychological and Social Well-Being of Childhood Cancer Survivors. Eur. J. Oncol. Nurs. Off. J. Eur. Oncol. Nurs. Soc..

[3] Wakefield C.E., McLoone J., Goodenough B., Lenthen K., Cairns D.R., Cohn R.J. (2010). The Psychosocial Impact of Completing Childhood Cancer Treatment: A Systematic Review of the Literature. J. Pediatr. Psychol..

[4] Tremolada M., Taverna L., Bonichini S., Putti M.C., Pillon M., Biffi A. (2020). Health Locus of Control in Parents of Children with Leukemia and Associations with Their Life Perceptions and Depression Symptomatology. Children.

[5] Tremolada M., Bonichini S., Basso G., Pillon M. (2018). Adolescent and Young Adult Cancer Survivors Narrate Their Stories: Predictive Model of Their Personal Growth and Their Follow-up Acceptance. Eur. J. Oncol. Nurs. Off. J. Eur. Oncol. Nurs. Soc..

[6] Duffey-Lind E.C., O’Holleran E., Healey M., Vettese M., Diller L., Park E.R. (2006). Transitioning to Survivorship: A Pilot Study. J. Pediatr. Oncol. Nurs..

[7] von Essen L., Enskär K., Kreuger A., Larsson B., Sjödén P.O. (2000). Self-Esteem, Depression and Anxiety among Swedish Children and Adolescents on and off Cancer Treatment. Acta Paediatr. Oslo Nor. 1992.

[8] Maggiolini A., Grassi R., Adamoli L., Corbetta A., Charmet G.P., Provantini K., Fraschini D., Jankovic M., Lia R., Spinetta J. (2000). Self-Image of Adolescent Survivors of Long-Term Childhood Leukemia. J. Pediatr. Hematol. Oncol..

[9] Tremolada M., Bonichini S., Taverna L. (2016). Coping Strategies and Perceived Support in Adolescents and Young Adults: Predictive Model of Self-Reported Cognitive and Mood Problems. Psychology.

[10] Barrera M., Shaw A.K., Speechley K.N., Maunsell E., Pogany L. (2005). Educational and Social Late Effects of Childhood Cancer and Related Clinical, Personal, and Familial Characteristics. Cancer.

[11] Bessell A.G. (2001). Children Surviving Cancer: Psychosocial Adjustment, Quality of Life, and School Experiences. Except. Child..

[12] Suppiah R., Patton M.A., McGrath P. (2005). Re-Entering Life: Paediatric Acute Myeloid Leukaemia at One Year Post Treatment. Aust. J. Holist. Nurs..

[13] Mattsson E., Lindgren B., Essen L.V. (2008). Are There Any Positive Consequences of Childhood Cancer? A Review of the Literature. Acta Oncol..

[14] Foster R.H., Russell C.C.R., Dillon R., Bitsko M.J., Godder K., Stern M. (2014). Relations Among Optimism, Perceived Health Vulnerability, and Academic, Self-Regulatory, and Social Self-Efficacy in Adolescent Survivors of Childhood Cancer. J. Psychosoc. Oncol..

[15] Caprara G.V., Fida R., Vecchione M., Del Bove G., Vecchio G.M., Barbaranelli C., Bandura A. (2008). Longitudinal Analysis of the Role of Perceived Self-Efficacy for Self-Regulated Learning in Academic Continuance and Achievement. J. Educ. Psychol..

[16] Pendley J.S., Dahlquist L.M., Dreyer Z. (1997). Body Image and Psychosocial Adjustment in Adolescent Cancer Survivors. J. Pediatr. Psychol..

[17] Fan S., Eiser C. (2009). Body Image of Children and Adolescents with Cancer: A Systematic Review. Body Image.

[18] Zeltzer L.K., Recklitis C., Buchbinder D., Zebrack B., Casillas J., Tsao J.C.I., Lu Q., Krull K. (2009). Psychological Status in Childhood Cancer Survivors: A Report from the Childhood Cancer Survivor Study. J. Clin. Oncol. Off. J. Am. Soc. Clin. Oncol..

[19] Tremolada M., Bonichini S., Weisner T., Basso G., Pillon M. (2013). Parental Narratives of Children with Leukemia in the Second Week after the Diagnosis: The Ecocultural Family Interview-Cancer. J. Pediatr. Oncol..

[20] Axia G., Weisner T. (2000). La Valutazione Dell’ecocultura Famigliare. La Valutazione Del Bambino.

[21] Connelly F.M., Clandinin D.J., Green J.L., Camili G., Elmore P.B. (2006). Narrative Inquiry. Handbook of Complementary Methods in Educational Research.

[22] Zannini L., Gambacorti-Passerini M.B. (2016). Coherence between data gathering technique and data analysis method in qualitative studies. A research experience based on leukemia survivors’ narratives. Encyclopaideia.

[23] De Clercq E., Elger B.S., Wangmo T. (2017). Missing life stories. The narratives of palliative patients, parents and physicians in paediatric oncology. Eur J. Cancer Care..

[24] Kahalley L.S., Conklin H.M., Tyc V.L., Wilson S.J., Hinds P.S., Wu S., Xiong X., Hudson M.M. (2011). ADHD and Secondary ADHD Criteria Fail to Identify Many At-Risk Survivors of Pediatric ALL and Brain Tumor. Pediatr. Blood Cancer.

